# Inhomogeneous Canham–Helfrich Abscission in Catenoid Necks under Critical Membrane Mosaicity

**DOI:** 10.3390/membranes13090796

**Published:** 2023-09-14

**Authors:** José Antonio Santiago, Francisco Monroy

**Affiliations:** 1 Departamento de Matemáticas Aplicadas y Sistemas, Universidad Autónoma Metropolitana Cuajimalpa, Vasco de Quiroga 4871, Ciudad de México 05384, Mexico; 2 Departamento de Química Física, Universidad Complutense de Madrid, Av. Complutense s/n, 28040 Madrid, Spain; monroy@quim.ucm.es; 3 Translational Biophysics, Institute for Biomedical Research, Hospital Doce de Octubre (imas12), Av. Andalucía s/n, 28041 Madrid, Spain

**Keywords:** inhomogeneous spontaneous curvature, catenoidal necks, stress on curved fluid membranes

## Abstract

The mechanical effects of membrane compositional inhomogeneities are analyzed in a process analogous to neck formation in cellular membranes. We cast on the Canham–Helfrich model of fluid membranes with both the spontaneous curvature and the surface tension being non-homogeneous functions along the cell membrane. The inhomogeneous distribution of necking forces is determined by the equilibrium mechanical equations and the boundary conditions as considered in the axisymmetric setting compatible with the necking process. To establish the role played by mechanical inhomogeneity, we focus on the catenoid, a surface of zero mean curvature. Analytic solutions are shown to exist for the spontaneous curvature and the constrictive forces in terms of the border radii. Our theoretical analysis shows that the inhomogeneous distribution of spontaneous curvature in a mosaic-like neck constrictional forces potentially contributes to the membrane scission under minimized work in living cells.

## 1. Introduction

Mitotic cell division, budding, endocytosis organelle fission and fusion, viral egress and bacterial fission are metabolically forced biological processes that follow formation of scissional necks connecting membrane splitting compartments [1,2,3,4,5,6,7]. These mechanical cell shaping processes are concomitant with compositional remodeling under cytokinetic action [8,9,10,11,12,13]. Despite the biological diversity beneath, functional conditions for fluid mosaicity, lateral incompressibility and bending elasticity are shared by the supporting lipid membrane [14,15,16,17,18]. Early evidence showed that membrane necking is tightly regulated under inhomogeneous traffic of lipids and proteins [1,2,3,19,20,21,22,23,24,25]. Critical phase transitions have been also revealed to lead functional membrane mosaicity [1,19,20,21,22,23,24,25]. More specifically, compositional inhomogeneities facilitate membrane necking along curvature pathways coordinated with the cellular membrane factories [20,21,22,23,26,27]. Further evidence has shown scissional membrane necks adapting compositional criticality under cytokinetic stresses [1,17,18,19,28,29]. Furthermore, membrane compositional heterogeneities have been also shown to have a key regulatory role under curvature generation [5,20,30]. Forced oscillations in necking asymmetry have also been observed in artificial vesicle dumbbells to be driven by active proteins that are able to change membrane curvatures [31,32]. Therefore, we theorize that constrictional necking is a compositional inhomogeneous process of membrane remodeling along critical bending pathways that are driven by highly directional tensions under subsidiary or negligible influence of hydrostatic forces releasing volume [33,34,35]. We also theorize that material distributions considered to be inhomogeneous can make areas vary anisotropically [4,8]. Those theoretical provisions could reasonably capture the biological idea of homeostatic fitness under mechanical equilibrium, i.e., they recapitulate Cannon’s concept of homeostasis as “physiological self-regulation able to maintain stability at each instantaneous configuration while adjusting the system to changing external conditions” [36]. Albeit that geometric and constitutional relationships between neck curvatures and membrane stresses have been explored for mechanically homogeneous necks and aare considered compositionally isotropic [5,6,37], a fundamental theory of inhomogeneous necking lead by compositional asymmetries is still lacking.

In this work, we model scissional necks under locally inhomogeneous membrane stresses at global dependence on two isotropic (fluidlike) elasticity parameters: (i) the bending rigidity (κ), controlling the mean (extrinsic) curvature $K=C_{1}+C_{2}$ (being $C_{i}$ the principal curvatures); and (ii) the saddle-splay modulus ($\kappa_{G}$), which couples the Gaussian curvature $K_{G}=C_{1}C_{2}$. We rely on the generalized version of the Canham–Helfrich (CH) theory for the free energy of the membrane deformations [38,39]:

(1) $$H=\frac{\kappa }{2}\int dA{\left(K-K_{0}\right)}^{2}+\kappa_{G}\int dA\;K_{G}+\sigma \int dA$$

where necking activity is considered in a field of curvature elasticity under spontaneous curvature ($K_{0}$), which further relates to membrane tension (σ). In this homogeneous CH functional, dA is the area element that describes an incompressible membrane.

Our necking model is assumed to resemble a deformable catenoid neck under anisotro-pic stress distributions due to axisymmetric deformations along the relevant parallel and meridian directions [40,41].

**Figure 1:**
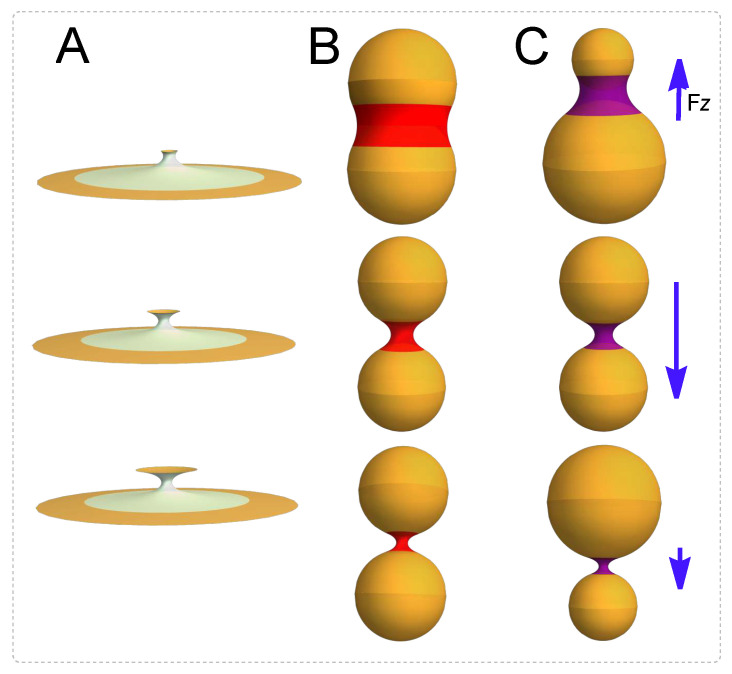
Membrane neck dynamics under scissional forces. (**A**) A necking process describing the membrane necks connecting scissional compartments. Two modes of membrane necking are possible. (**B**) Symmetric mode: two spherical membranes compartments of equal radii joined by a catenoidal neck in a symmetric process. (**C**) Asymmetric mode: membrane compartment of different radii joined by an asymmetric catenoid.

We have studied the effect of $K_{0}$-inhomogeneities on the mechanical anisotropies of catenoidal membranes. The curvature–covariant framework is exploited to analyze some biological necking processes as depicted in Figure 1. We have obtained the interaction force between the catenoid boundaries and then focussed on the mechanical balance along the axial and radial directions. The presence of a constant external field of neck stretching $\eta =-F_{z}$ balances the axial force $F_{z}$, whilst the radial balance requires a constrictional external field variable along the *z*-coordinate, $\gamma =-F_{\rho }$. In a typical necking abscission process like the one shown in Figure 1, there is a critical catenoid such that the axial force η requests an abrupt change from being a positive tension to a negative one. This change results in a compositional switching for the distribution of the spontaneous curvature, $K_{0}$. If the necking process is symmetric, as illustrated in column B, the radial force between the borders has been shown to undergo either repulsive or attractive interactions akin to a diatomic molecule. For small relative radii corresponding to incipient constriction (thick catenoid), this interaction appears repulsive. In the region with relative large radii, the radial force becomes attractive. Finally, for the larger radii corresponding to higher pinching constrictions and for larger radii (thin catenoid), the borders have no interaction. Whereas the critical catenoid and the catenoid of maximum area are the same geometric object in symmetric necking (Figure 1B), smaller membrane area reorganizations are involved in the asymmetric cases (see Figure 1C). As a further insight in the covariant mechanics of abscissional membrane catenoids, we describe the geometric criticality behind such inhomogeneous necking physics.

## 2. Methods

To capture the functional principle of homeostatic fitness under instantaneously reversible mechanical equilibrium, our mean-field CH reductionism recapitulates particular necking conditions of adaptative slowness (staticity) and absence of frictional dissipation (fluidity), with both leading adiabatic (isoentropic) stability compatible with physiological homeostasis [42]. Below, we implement an inhomogeneous CH model as being an optimaly homeostatic (conservative) expenditure of elastic free energy for membrane shape remodeling under quasi-static necking. In general, the CH model does not explicitly take possible chemical change requested under elastic deformation into account—neither does it consider entropy creation related to material change [39,43]. However, we effectively consider a heterogeneous class of chemical potential as embedded within the anisotropic membrane tension inhomogeneity [5,6,44]. We also consider the spontaneous curvature being laterally inhomogeneous, thus capturing the idea of optimized shape remodeling under local curvature effection [6,36,45]. In biological terms, inhomogeneous sigma and $K_{0}$ imply introducing mesoscopic stressors of local flexibility effectively imparted under lateral membrane mosaicity. The global neck rigidness is recapitulated under homogeneous rigidities for pure splay (κ) and saddle-splay ($\kappa_{G}$), with fluidlike properties naturally deployed along the membrane shape. They constitute intensive densities of elastic resistance isotropically fixed under rotational symmetry (mesoscopic flexible fluidness property invoked by W. Helfrich in his foundational formulation of the model [39]. Indeed, κ and $\kappa_{G}$ both fix the global amount of energy adiabatically conserved by the CH system. Based on the mesoscopically inhomogeneous CH mean-field of curvature elasticity, we will describe the necking process as quasi-static sequences of equilibrium configurations considered to be adiabatically stable (homeostatic) as being energetically optimal (no heat exchange involved), i.e., not exhibiting dissipation in a near-reversible (frictionless) succession of mechanically equilibrated fluid states [42].

*Inhomogeneous elastic energy*. As defined upon the Canham–Helfrich functional describing mean-field curvature elasticity [38,39], for the inhomogeneous case, we have a state:

(2) $$H_{inhom}=\frac{\kappa }{2}\int dA{\left(K-K_{0}\right)}^{2}+\kappa_{G}\int dA\;K_{G}+\int dA\;\sigma +P\int dV,$$

where κ and $\kappa_{G}$ represent the constant (globally averaged) values of the flexural rigidities for, respectively, bending and saddle-splay modes. Here, dV is the differential volume element (the integrated volume is enforced under hydrostatic pressure *P*). Both the spontaneous curvature ($K_{0}$) and the surface tension (σ) are considered inhomogeneous functions of the membrane coordinates, i.e., they are variable quantities that depend on the local stresses. As a strong condition for membrane heterogeneity, the local surface tension σ has been introduced as a coordinate-dependent Lagrange multiplier, which locally fixes the surface area of each membrane element. Conceptually, σ is an inhomogeneous energy density considered to be chemically open and encoding an anisotropic distribution of membrane tensions (axial stretching η and radial constriction γ). This is different to the usually isotropic membrane tension describing the close chemical equilibrium with a membrane reservoir [5,46]. However, the Gaussian term determined by $K_{G}$ is always considered as a conserved quantity involved until complete scission is explicitly considered (it remains constant without topological change: Gauss–Bonnet theorem [47]). This topological invariant becomes chiefly relevant into the boundary terms injecting curvature energy for necking.

*Mean-field parameters: microphysical interpretation*. The CH model is built by coarse-graining the microscopic details on the interactions at the molecular level in the membrane. Indeed, part of its success lies in the fact that it is a phenomenological model that describes many of the configurations and elastic forces observed in fluid membranes at the mesoscopic level [43]. The descriptive mesoscopic scale of the CH model implies a two-dimensional surface that does not take into account the membrane thickness nor their internal complexities which are recapitulated in an effective flexible and globally fluid surface sheet. The microscopic compacting interactions are globally averaged at the molecular level; thus, they are effectively projected in the elastic parameters and considered to be globally homogeneous as constant values κ and $\kappa_{G}$ [44,48]. For instance, spontaneous membrane bending can be locally induced either by intrinsic transverse asymmetry leading to curvature remodeling (e.g., by inserted proteins, curvature shaping lipids with a molecular conical shape, asymmetric electrostatics, etc.) [20,21,22,23,24] or by extrinsic effectors of lateral asymmetry (e.g., cortical flows) leading to preferred curvature that balances torques due to differential stress and bending moments in each side of the membrane [45].

The microphysical origin of the local membrane asymmetries is recapitulated under the pressure profiles along the internal coordinates (lateral and transverse) [36,49]. When a transversely asymmetric membrane is curved, the normal unit vector can be nonuniform in each leaflet, so that that the moments of the stress depend on the lateral coordinates. Hence, it implies that the curvature effectors are non-homogeneous functions along the membrane [45,50]. The lateral nonuniformness in the stress profile could introduce further complexity and become intercoupled with the local torques [40]. In order to simplify the mesoscopic analysis, our current work assumes that the local asymmetries in the curved membrane are only manifested in the longitudinal coordinate through an inhomogeneous spontaneous curvature $K_{0}\left(l\right)$ and anisotropic surface tension σ(l), considered as an inhomogeneous constraint to local area. Both inhomogeneous properties are expected to be locally intercoupled [51], indicating locally compressed, curved regions in the floppy case (if σ(l)<0) and stretched, flatter regions in the tensioned case (if σ(l)>0). Furthermore, the effective in-plane membrane stresses can be seen as the longitudinal imbalance between the two leaflets in such a way that the global stress results in some preferential curvature in the membrane [45]. The thicker the membrane in relation to curvature, the higher the induced torque necessary to cancel out the stress [52]. We later also included the hydrostatic pressure to recapitulate the isotropic stress of the bulk cytoplasm (*P*). It has been considered as a Lagrange multiplier to enforce the volume constraint in the closed vesicle configurations used to develop the surface covariant theory. In the particular case of an open surface, e.g., a membrane neck, then P=0. All these microphysical complexities have been epitomized under the systemic parameters of the inhomogeneous CH model above described in Equation (2).

*Surface geometry*. A generic surface in $R^{3}$, with cartesian coordinates $x=(x^{1},x^{2},x^{3})$, can be parametrized by the embedding function $x=X(u^{a})$, where $u^{a}$ are local coordinates on the surface (a=1,2). The infinitesimal 3D-Euclidean distance $ds^{2}=dx\cdot dx$ induces the corresponding arc length distance on the surface $ds^{2}=g_{ab}du^{a}du^{b}$; here, $g_{ab}:=e_{a}\cdot e_{b}$ is the induced metric, and $e_{a}:=\partial_{a}X$ are two local tangent vector fields. Correspondingly, the induced metric defines a covariant derivative on the surface denoted by $\nabla_{a}$. The unit normal to the surface is $n\;=\left(\varepsilon^{ab}/2\right)e_{a}\times e_{b}$, where $\varepsilon^{ab}=\epsilon^{ab}/\sqrt{g}$ with $\epsilon^{ab}$ being the Levi–Civita alternating symbol and $g=\det\left(g_{ab}\right)$. The Gauss equation establishes $\nabla_{a}e_{b}=-K_{ab}n$, which describes the change of the tangent vector fields along the surface. The components of the extrinsic curvature are defined as $K_{ab}:=-\nabla_{a}e_{b}\cdot n$, which are related with the Gaussian curvature $K_{G}$ through of the Gauss–Codazzi equation, $K_{a}^{c}K_{cb}=KK_{cb}-g_{ab}K_{G}$, and its contraction $K^{ab}K_{ab}=K^{2}-2K_{G}$ [47]. The Codazzi–Mainardi equation $\nabla_{a}K^{ab}=\nabla^{b}K$, will be also useful.

*Stress tensor.* Under an infinitesimal surface deformation, δX, the variation of the energy, is written as $\delta H=\int \;dA\nabla_{a}f^{a}\cdot \delta X$, where $f^{a}$ is the stress tensor [40,41]:

(3) $$f^{a}=f^{ab}e_{b}+f^{a}n,$$

with tangential and normal components, respectively:

(4) $$\begin{array}{ccc} f^{ab} & = & \kappa \left(K-K_{0}\right)\left[K^{ab}-\frac{1}{2}\left(K-K_{0}\right)g^{ab}\right]-g^{ab}\sigma ,\\ f^{a} & = & -\kappa \nabla^{a}\left(K-K_{0}\right). \end{array}$$

For closed membranes (spherical topology), the hydrostatic pressure term −PV is considered within the energy in Equation (2); the difference $P=P_{in}-P_{out}$ is the pressure jump that supports the membrane vesicle, and *V* is the enclosed volume. For closed (topologically spherelike) cells in mechanical equilibrium, the surface divergence of the stress tensor is hence found as

(5) $$\nabla_{a}f^{a}=P\;n.$$

Therefore, substituting Equation (3) into Equation (5), the equilibrium conditions can be reestablished in terms of the stresses in Equation (4) as

(6) $$\begin{array}{ccc} \nabla_{a}f^{a}-K_{ab}f^{ab} & = & P,\\ \nabla_{a}f^{ab}+f^{a}K_{a}{}^{b} & = & 0. \end{array}$$

These equilibrium equations define the generalized theoretical framework for calculating constitutional relationships corresponding to the inhomogeneous mosaic-like membrane. They determine the membrane shape in terms of locally inhomogeneous elasticity (as given by variable elastic parameters).

*Inhomogeneous CH membrane: local shape equations.* Specifically, Equation (4) provides analytic expressions for the local stresses compatible with the inhomogeneous CH membrane, as considered at global mechanical equilibrium, i.e., under $\delta H_{inhom}=0$. By substituting particular expressions of Equation (4) in the generalized equilibrium equations of Equation (6), one immediately obtains the inhomogeneous connections between local elasticity and curvatures.

The first condition in (6) accounts for mechanical stability along the normal direction:

(7) $$\begin{array}{c} -\kappa \nabla^{2}\left(K-K_{0}\right)-\frac{\kappa }{2}\left(K-K_{0}\right)\left[K\left(K+K_{0}\right)-4K_{G}\right]+\sigma K=P, \end{array}$$

which describes the connection between local shape and Laplace pressure at any point in the membrane. In the homogeneous case (for constant σ and $K_{0}$), Equation (7) reduces to the well-known Helfrich’s shape equation [53,54].

The second condition in Equation (6) accounts for the lateral equilibrium relationship, or equivalently stated, as an inhomogeneity connection between the spontaneous curvature and the tensile stress in the tangent plane through of the geometric shape; that is,

(8) $$\partial_{a}\sigma =\kappa \left(K-K_{0}\right)\partial_{a}K_{0}.$$

This result is especially interesting as it connects the changes of constitutive properties in a causal relationship modulated by the local value of mean curvature. Being completely novel in the analytical form here presented, the concept is also quite intuitive in biological terms. It is hence useful for interpreting the mechanical impact of compositional agents for mosaic curvature making.

*Closed vesicles: directional stresses.* After integrating Equation (5) in a closed geometry, by taking advantage of the divergence theorem, we obtain:

(9) $$\oint_{C}\;ds\;f^{a}l_{a}=P\;\int_{M}dA\;n,$$

where *C* represents the boundary for an arbitrary contour for membrane patch M. The left hand side of Equation (9) represents the elastic force which the membrane domain M exerts onto the rest of the closed vesicle $M^{\prime }$. Nevertheless, this force is manifested on the membrane boundary *C*, independently of its particular shape and/or size changing under deformation. The right hand side states that, at equilibrium, this deformation work is equalized to the normal pressure flux across its membrane area. The integral is then calculated along the loop *C* with tangent $T=T^{a}e_{a}$, and conormal $l=l^{a}e_{a}$ is as defined in the Darboux frame adapted to the curve *C*.

In the Darboux basis, the force term in the left hand side of Equation (9) is

(10) $$f^{a}l_{a}=F_{T}T+F_{l}l+F_{n}n,$$

with separated elastic force, per unit length, along each relevant membrane degree of freedom; these are, respectively, tangential, normal and lateral, directions

(11) $$\begin{array}{ccc} F_{T} & \equiv & \kappa \left(K-K_{0}\right)K_{\tau },\; \end{array}$$

(12) $$\begin{array}{ccc} F_{n} & \equiv & -\kappa \nabla_{l}\left(K-K_{0}\right),\; \end{array}$$

(13) $$\begin{array}{ccc} F_{l} & \equiv & -\sigma +\frac{\kappa }{2}\left[K_{l}^{2}-{\left(K_{T}-K_{0}\right)}^{2}\right]. \end{array}$$

Here, the curvature gradients are expressed as $K_{l}\equiv K_{ab}l^{a}l^{b}$, $K_{T}\equiv K_{ab}T^{a}T^{b}$, $K_{\tau }\equiv K_{ab}l^{a}T^{b}$ and $\nabla_{l}K\equiv l^{a}\nabla_{a}K$.

Similarly, in order to calculate the force that a meridian region does exert on the adjacent side, we project the stress tensor on the meridian loop, i.e., $f^{a}T_{a}$. Then,

(14) $$f^{a}T_{a}=F_{T}^{m}T+F_{l}^{m}l+F_{n}^{m}n,$$

where the projections are

(15) $$\begin{array}{ccc} F_{T}^{m} & \equiv & -\sigma -\kappa \frac{K_{0}^{2}}{2}+\frac{\kappa }{2}\left(K_{T}^{2}-K_{l}^{2}+2K_{0}K_{l}\right), \end{array}$$

(16) $$\begin{array}{ccc} F_{n}^{m} & \equiv & -\kappa \nabla_{T}\left(K-K_{0}\right),\; \end{array}$$

(17) $$\begin{array}{ccc} F_{l}^{m} & \equiv & \kappa \left(K-K_{0}\right)K_{\tau },\; \end{array}$$

for the directional derivative along the tangent, $\nabla_{T}\equiv T^{a}\nabla_{a}$.

*Open membranes: boundary conditions.* For open membranes considered hydrostatically depressurized with respect to surrounding ambiance (P=0), the boundary conditions are given by [55]

(18) $$\begin{array}{ccc} \kappa (K-K_{0}) & = & -\kappa_{G}k_{n},\; \end{array}$$

(19) $$\begin{array}{ccc} \frac{\kappa }{2}{\left(K-K_{0}\right)}^{2}+\sigma & = & -\kappa_{G}K_{G}+\sigma_{b}k_{G}\; \end{array}$$

(20) $$\begin{array}{ccc} \kappa \nabla_{l}K & = & \kappa_{G}{\dot{K}}_{\tau }. \end{array}$$

The first condition in Equation (18) implies that, on the boundary edge, the torque is proportional to the projection of the extrinsic curvature, $K_{ab}$, on the tangent vector, $k_{n}\equiv K_{ab}T^{a}T^{b}$ (the normal curvature of the boundary curve); being the constant of proportionality, the relative Gaussian stiffness is referred to the bending modulus, $\lambda \equiv \kappa /\kappa_{G}$. The second boundary condition, Equation (19), identifies the local density of membrane energy on the border edge with the Gaussian curvature therein and involves the geodesic curvature of the border, $k_{G}$. Finally, the dot on $K_{\tau }$ in Equation (20) represents the derivative respect to the arc length of the boundary edge.

**Figure 2:**
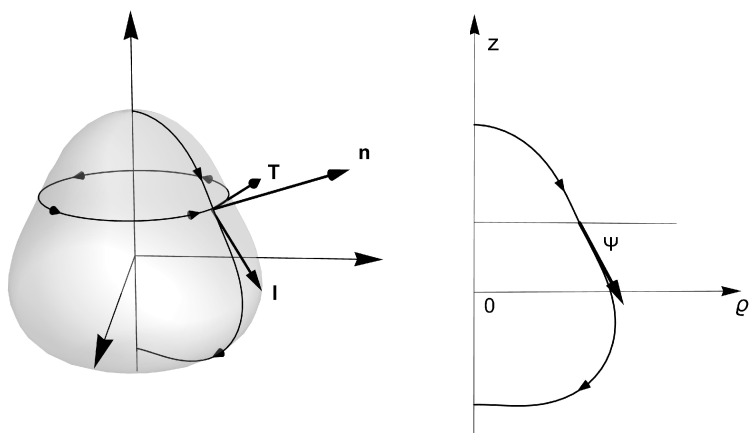
A generic closed surface of revolution. In cylindrical coordinates {ρ,ϕ,z}, it is described with the embedding function: X(l,ϕ)=ρ(l)ρ+z(l)k in the cylindrical basis ρ=(cosϕ,sinϕ,0), k=(0,0,1) and ϕ=(−sinϕ,cosϕ,0). The generating curve is parametrized with arc length *l*, so that ${\rho^{\prime }}^{2}+{z^{\prime }}^{2}=1$, while $\rho^{\prime }=\cos\Psi$ and $z^{\prime }=-\sin\Psi$, where Ψ is the tangential angle of the generating curve, and the derivative with respect to *l* is denoted as a ${}^{\prime }$. The unit normal can be written as n=sinΨρ+cosΨk. The unit tangent vector, adapted to parallels on axially symmetric surfaces, is given by T=ϕ, while the unit conormal is l=cosΨρ−sinΨk. The orthonormal set {T,l,n} constitutes the Darboux basis adapted to the parallel loop.

*Axial symmetry: surface membranes of revolution*. Axial membrane with cylindrical symmetry is described in Figure 2 and is parametrized in terms of the inclination angle (Ψ) and the radial coordinate (ρ). In these axisymmetric coordinates, the projection of the extrinsic curvature onto the conormal vector field l is given by the projection $K_{l}\equiv K_{ab}l^{a}l^{b}=\Psi^{\prime }$ (where the symbol ${}^{\prime }$ means the derivative respect to the arc length *l*). On the unit tangent along the parallels to the equatorial plane, we have the condition $K_{T}\equiv K_{ab}T^{a}T^{b}=\left(\sin\Psi \right)/\rho$, which indicates their flexural curvature. We have also the rotational projection normal to the revolution *z*-axis, i.e., $K_{\tau }\equiv K_{ab}l^{a}T^{b}=0$, which indicates that no change is needed for extrinsic rotational strain to generate the axially symmetric revolution surface. The Gaussian curvature is given by $K_{G}\equiv -\rho^{\prime \prime }/\rho =\Psi^{\prime }\left(\sin\Psi \right)/\rho$, which is the canonical parametrization for the intrinsic curvature (Gauss’ Theorem *Egregium*). This definition as the relative curvature of the parametric curve depends only on the intrinsic distances measured on the surface but not on how it is extrinsically embedded in Euclidean space. In terms of the tangential inclination angle Ψ, the (twice) mean curvature is given by $K=\Psi^{\prime }+\left(\sin\Psi \right)/\rho$. Therefore, the projections of the force in the Darboux frame, Equations (11), (12) and (13) are, respectively, given as

(21) $$\begin{array}{ccc} F_{T} & \equiv & 0,\; \end{array}$$

(22) $$\begin{array}{ccc} F_{n} & \equiv & -\kappa {\left(\Psi^{\prime }+\frac{\sin\Psi }{\rho }-K_{0}\right)}^{\prime },\; \end{array}$$

(23) $$\begin{array}{ccc} F_{l} & \equiv & -\sigma +\frac{\kappa }{2}\left[\Psi^{\prime 2}-{\left(\frac{\sin\Psi }{\rho }-K_{0}\right)}^{2}\right]. \end{array}$$

Similarly, in Equations (15), (16) and (17), the meridian projections can be identified as

(24) $$\begin{array}{ccc} F_{T}^{m} & \equiv & -\sigma +\frac{\kappa }{2}\left[\frac{\sin^{2}\Psi }{\rho^{2}}-{\left(\Psi^{\prime }-K_{0}\right)}^{2}\right], \end{array}$$

(25) $$\begin{array}{ccc} F_{n}^{m} & \equiv & 0,\; \end{array}$$

(26) $$\begin{array}{ccc} F_{l}^{m} & \equiv & 0.\; \end{array}$$

*Axial and radial forces acting through parallels.* By taking advantage of the axial symmetry, we project the stress tensor along the meridian generatrix l (see Figure 2). Consequently, the forces per unit length decomposed on the axial and the radial directions are

(27) $$\begin{array}{ccc} F_{z} & \equiv & f^{a}l_{a}\cdot k=\cos\Psi F_{n}-\sin\Psi F_{l}, \end{array}$$

(28) $$\begin{array}{ccc} F_{\rho } & \equiv & f^{a}l_{a}\cdot \rho =\sin\Psi F_{n}+\cos\Psi F_{l}, \end{array}$$

where $F_{n}$ and $F_{l}$ are given by Equations (22) and (23), respectively. Further, multiplying by the factor 2πρ, the corresponding necking forces are obtained (see below).

A further integration of Equation (5) within the axial open neck symmetry yields the mechanical balance equations on parallels and meridians. They are equivalent to the equilibrium equations obtained from the variational Lagrangian analysis in cylindrical coordinates [56].

## 3. Julicher–Seifert Variational Approach

As devoid of the conserved Gaussian term, the functional H in (2), can be rewritten in cylindrical coordinates as an action integrated over the generatrix parameter (*l*); for the considered axial symmetry, this corresponds to the arc length coordinate defined along the meridian. Consequently, the inhomogeneous action is given by [56]

(29) $$H_{inhom}=\int_{l_{1}}^{l_{2}}dlL_{inhom},$$

where the Lagrangian function is given by

(30) $$\begin{array}{ccc} L_{inhom} & = & \pi \rho \kappa {\left(\Psi^{\prime }+\frac{\sin\Psi }{\rho }-K_{0}\right)}^{2}+2\pi \sigma \rho +\gamma \left(\rho^{\prime }-\cos\Psi \right)\\ & + & \eta (z^{\prime }+\sin\Psi ), \end{array}$$

and the Lagrange multipliers γ and η have been added, so that the Euler–Lagrange equation with respect to radial constriction (γ) and axial stretching (η) holds, respectively, gaining values of $\rho^{\prime }=\cos\Psi$ and $z^{\prime }=-\sin\Psi$. Further:

(i) Euler–Lagrange equation respect to the inclination angle Ψ, gives
  (31) $$2\pi \rho \;F_{n}+\left(\gamma \sin\Psi +\eta \cos\Psi \right)=0,$$
  where we have considered Equation (22). Hence, Equation (31) corresponds to the mechanical balance along the unit normal to the surface; note that the term between parentheses actually corresponds to the projection of the constrictive force, λ=γρ+ηk, along the unit normal, i.e., Equation (31) may be rewritten as $2\pi \rho \;F_{n}+\lambda \cdot n=0$, which indicates the mechanical balance between normal stress and necking constriction.
(ii) Euler–Lagrange equation with respect to ρ obtains
  (32) $$\begin{array}{ccc} \gamma^{\prime } & = & \pi \kappa \left[{\left(\Psi^{\prime }-K_{0}\right)}^{2}-\frac{\sin^{2}\Psi }{\rho^{2}}\right]+2\pi \sigma ,\\ & = & -2\pi F_{T}^{m}, \end{array}$$
  where we have substituted Equation (24) in order to obtain the final form in the second line. This equation corresponds to the balance of forces, per unit length, that acts through meridians along the tangential direction.
(iii) Euler–Lagrange with respect to *z* is given by $\eta^{\prime }=0$ so that η is a constant. This result is a consequence of the Lagrangian symmetry on translations along the axial direction. This is the analogue of Equation (32) on the axial direction,
  (33) $$\begin{array}{ccc} \eta^{\prime } & = & -2\pi F_{l}^{m},\\ & = & 0, \end{array}$$
  where we used Equation (26).
(iv) Since the parameter *l* does not appear explicitly in the Lagrangian, *L*, the Hamiltonian function is $H\equiv -L_{inhom}+\Psi^{\prime }\partial L/\partial \Psi^{\prime }+\rho^{\prime }\partial L/\partial \rho^{\prime }+z^{\prime }\partial L/\partial z^{\prime }=0$ along the membrane [56]. As a consequence,
  (34) $$2\pi \rho F_{l}+\left(\gamma \cos\Psi -\eta \sin\Psi \right)=0,$$
  where we have used Equation (23). This equation represents the balance of forces along the lateral direction. The term in parentheses is the projections of the constrictive force, λ=γρ+ηk, as calculated along the unit conormal l, i.e., Equation (34) can be expressed as $2\pi \rho F_{l}+\lambda \cdot l=0.$

Finally, in cylindrical coordinates, Equation (8) is restated as

(35) $$\sigma^{\prime }=\kappa \left(\Psi^{\prime }+\frac{\sin\Psi }{\rho }-K_{0}\right)K_{0}^{\prime }.$$

Thus, the relationship between the balance equations and the Euler–Lagrange equations has been clarified in the natural coordinates for describing the necking process and further generalized to the inhomogeneous case. As a relevant example in the focus of this paper, let us analyze the necking case of the catenoid, which is a minimal surface that implies analytical solutions for the spontaneous curvature.

## 4. Results: Catenoidal Necks

**Figure 3:**
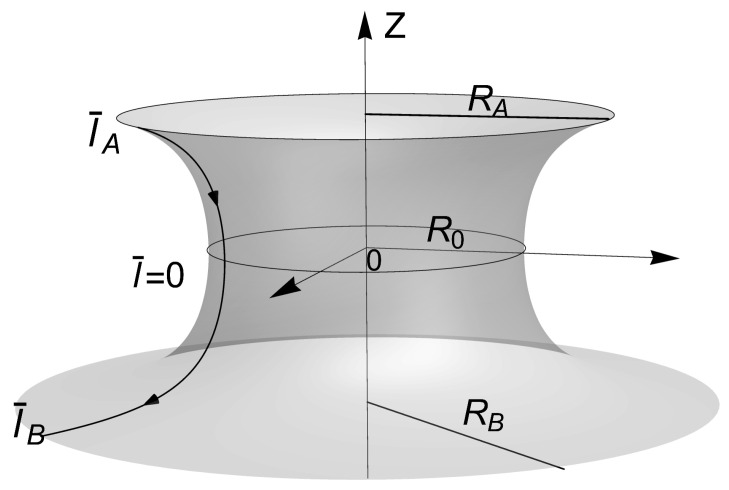
Definition of the catenoid in terms of the equatorial radii (R) and axial length (L); $\bar{\rho }\equiv \rho /R_{0}$, $\bar{z}\equiv z/R_{0}$. The equation of the meridian catenary generator with fixed neck radii $R_{0}$ is given by $\bar{\rho }=\cosh\bar{z}$. The catenoid can thus be re-parametrized in terms of the reduced arc length $\bar{l}\equiv l/R_{0}$, through the functions $\bar{\rho }=\sqrt{1+{\bar{l}}^{2}}$ and $\bar{z}=-\mathrm{arcsinh}\;\bar{l}$; in the upper border ${\bar{l}}_{A}=-\sqrt{{\bar{R}}_{A}^{2}-1}$, on the equatorial site, $\bar{l}=0$, and in the lower border ${\bar{l}}_{B}=\sqrt{{\bar{R}}_{B}^{2}-1}$. The relationship with the tangential angle of the generating curve: $\sin\Psi =1/\sqrt{1+{\bar{l}}^{2}}$, and $\cos\Psi =\bar{l}/\sqrt{1+{\bar{l}}^{2}}$. The derivative respect to $\bar{l}$ is given by ${\bar{\Psi }}^{\prime }=-1/\left(1+{\bar{l}}^{2}\right)$. The area of each hemisphere is rescaled in terms of its corresponding border; thus, the rescaled surface area ${\bar{A}}_{i}\equiv A_{i}/\left(2\pi R_{i}^{2}\right)$ in the upper hemisphere, e.g., is given by $2{\bar{A}}_{i}\left({\bar{R}}_{i}\right)=\sqrt{{\bar{R}}_{i}^{2}-1}/{\bar{R}}_{i}+{\bar{R}}_{i}^{-2}\mathrm{arctanh}\left(\sqrt{{\bar{R}}_{i}^{2}-1}/{\bar{R}}_{i}^{2}\right)$, where ${\bar{R}}_{i}=\left\{{\bar{R}}_{A},{\bar{R}}_{B}\right\}$. Similarly, the height is $\bar{h}={\bar{R}}_{A}^{-1}\mathrm{arcsinh}\left(\sqrt{{\bar{R}}_{A}^{2}-1}\right)+{\bar{R}}_{B}^{-1}\mathrm{arcsinh}\left(\sqrt{{\bar{R}}_{B}^{2}-1}\right)$.

*Equilibrium necking equations.* Figure 3 depicts a generalized axial neck symmetry, implying that the systemic equations do not depend on the azimuthal angle around the parallels but on the arc length parameter (*l* as describing neck latitudes in the generating meridian); this generatrix is mechanically inhomogeneous with origin at the neck equator (l=0; see caption for details). The catenoid is a minimal surface, such that the mean curvature is zero elsewhere (K=0). Therefore, the analytic integration of the covariant differential equation that connects membrane tension and spontaneous curvature, Equation (35), gives the conservation equation

(36) $$\bar{\sigma }\left(\bar{l}\right)+{\bar{K}}_{0}^{2}\left(\bar{l}\right)/2=C_{0},$$

where $\bar{\sigma }\equiv \sigma R_{0}^{2}/\kappa$, ${\bar{K}}_{0}\equiv R_{0}K_{0}$ and $\bar{l}\equiv l/R_{0}$, with $C_{0}$ being a constant of integration. This can be determined by the boundary condition, Equation (19), as $C_{0}=-\left(\kappa_{G}/\kappa \right){\bar{K}}_{G}\left({\bar{l}}_{A}\right)=\left(\kappa_{G}/\kappa \right){\bar{R}}_{A}^{-4}$, where ${\bar{l}}_{A}$ denotes the most northern latitude at the upper boundary (see Figure 3).

Therefore, the energy density is determined by the Gaussian curvature, $K_{G}$, at the boundary. In dimensionless variables, the mechanical balance along the axial and radial directions is obtained by using Equations (28), (31) and (34); the corresponding equilibrium equations are given, respectively, by

(37) $$\begin{array}{c} \bar{\rho }\cos\Psi {\bar{K}}_{0}^{\prime }+\bar{\rho }\sin\Psi \;\Psi^{\prime }K_{0}+C_{0}\bar{\rho }\sin\Psi =-\bar{\eta }, \end{array}$$

(38) $$\begin{array}{c} \bar{\rho }\sin\Psi {\bar{K}}_{0}^{\prime }-\bar{\rho }\cos\Psi \;\Psi^{\prime }{\bar{K}}_{0}-C_{0}\bar{\rho }\cos\Psi =-\bar{\gamma }, \end{array}$$

where $\bar{\rho }\equiv \rho /R_{0}$, $\bar{\eta }\equiv \eta R_{0}/\left(2\pi \kappa \right)$, and $\bar{\gamma }\equiv \gamma R_{0}/\left(2\pi \kappa \right)$.

By using the parametric equation of the catenoid (see the caption in Figure 3), the axial and radial mechanical balance on any parallel curve of the catenoid (see Figure 3) holds, respectively:

(39) $$\begin{array}{ccc} F_{z} & \equiv & \bar{l}{\bar{K}}_{0}^{\prime }\left(\bar{l}\right)-\frac{{\bar{K}}_{0}\left(\bar{l}\right)}{1+{\bar{l}}^{2}}+\frac{1}{\lambda }\frac{1}{{\bar{R}}_{A}^{4}}=-\bar{\eta },\; \end{array}$$

(40) $$\begin{array}{ccc} F_{\rho } & \equiv & {\bar{K}}_{0}^{\prime }\left(\bar{l}\right)+\frac{\bar{l}}{1+{\bar{l}}^{2}}{\bar{K}}_{0}\left(\bar{l}\right)-\frac{1}{\lambda }\frac{\bar{l}}{{\bar{R}}_{A}^{4}}=-\bar{\gamma }\left(\bar{l}\right). \end{array}$$

where the scaled elastic forces are along the axial and the radial direction as the relevant necking forces under the membrane tension; these are $\bar{\eta }=-F_{z}$ for the dimensionless stretching tension (considered constant and homogeneous in order to preserve the axial symmetry under translations) and $\bar{\gamma }\left(\bar{l}\right)=-F_{\rho }$ for the radial constrictive tension (considered essentially inhomogeneous).

By opposition to the homogeneous case (when ${\bar{K}}_{0}$ is constant), the meridian gradient of spontaneous curvature is herein identified to be a normal force that a region bounded by a parallel exerts on an adjacent region. Thus, in both Equations (39) and (40), the first term in the left hand side is the contribution of the normal force, whereas the next two terms are the lateral forces acting through the parallel of coordinate $\bar{l}$. Evaluating Equation (40) at the equatorial loop, $\bar{l}=0$, gives the constrictive necking force as the local change of spontaneous curvature, i.e., $C\equiv {\bar{K}}_{0}^{\prime }\left(0\right)$; thus, in the case of a symmetric distribution of ${\bar{K}}_{0}\left(\bar{l}\right)$ along the membrane, the force C=0. According to Equation (40), in the symmetric case, the scaled radial force, $-\bar{\gamma }\left(\bar{l}\right)$, is an odd function of the arc length parameter $\bar{l}$, and Equation (39) holds the relationship between the axial force and the spontaneous curvature at the waist of the catenoid; this is $-\bar{\eta }=-{\bar{K}}_{0}\left(0\right)+C_{0}$. Although in the symmetric case, the constrictive force vanishes, i.e., C=0, the scaled local torque has the maximum intensity at the waist of the neck; this particular property happens because the torque, $\bar{m}\left(\bar{l}\right)\equiv mR_{0}/\kappa$ (associated with local flexures around the loop) involves the spontaneous curvature itself [40]

(41) $$\bar{m}\left(\bar{l}\right)=\left({\bar{K}}_{0}\left(\bar{l}\right)-\frac{1}{\lambda (1+{\bar{l}}^{2})}\right)T.$$

In general, the inhomogeneous spontaneous curvature does appear to be a contribution to the net torque arising for flexural neck shaping, whereas its local change as a normal force (due to inhomogeneity) determines the constrictional stresses emerging from the equilibrated equator, i.e., $C\left(0\right)\equiv K_{0}^{\prime }\left(0\right)$. As an original result of this work, below, we obtain the necking solutions for inhomogeneous catenoids.

### 4.1. Inhomogeneous Solutions: Necking Forces

Once the balance equations have been established into the relevant directions, we obtain solutions for the inhomogeneous spontaneous curvature ${\bar{K}}_{0}$. These are

(42) $$\lambda {\bar{K}}_{0}\left(\bar{l}\right)=\left(\lambda \bar{\eta }+\frac{1}{{\bar{R}}_{A}^{4}}\right)S\left(\bar{l}\right)+\frac{\lambda C\;\bar{l}}{\sqrt{1+{\bar{l}}^{2}}},$$

as written in terms of the even function $S\left(\bar{l}\right)\equiv 1-\left(\bar{l}/\sqrt{1+{\bar{l}}^{2}}\right)\mathrm{arctanh}\left(\bar{l}/\sqrt{1+{\bar{l}}^{2}}\right).$ The constrictive force C appears here naturally as an integration constant for the spontaneous curvature force. On the border of the catenoid, we may write the function $S\left({\bar{R}}_{i}\right)=1-\left(\sqrt{{\bar{R}}_{i}^{2}-1}/{\bar{R}}_{i}\right)\mathrm{arctanh}\left(\sqrt{{\bar{R}}_{i}^{2}-1}/{\bar{R}}_{i}\right)$, where i={A,B} (see Figure 3). Solutions in Equation (42) are modulated by constitutive forces referred to the relative elasticity parameter $\lambda \equiv \kappa /\kappa_{G}$, defined as a control parameter for retaining further bending on the natural saddle-splay of the catenoid neck; these solutions are (i) the bending-rescaled axial tension ($\lambda \bar{\eta }$); and (ii) the border spontaneous curvature stress as recapitulated by C. The boundary conditions ${\bar{K}}_{0}\left({\bar{l}}_{i}\right)=\lambda^{-1}{\bar{R}}_{i}^{-2}$ [55] yield the bounding values of spontaneous curvature from which the constitutive parameters λC and $\lambda \bar{\eta }$ can be found as

(43) $$\begin{array}{ccc} \lambda C({\bar{R}}_{A},{\bar{R}}_{B}) & = & \frac{{\bar{R}}_{A}^{2}S\left({\bar{R}}_{A}\right)-{\bar{R}}_{B}^{2}S\left({\bar{R}}_{B}\right)}{{\bar{R}}_{A}{\bar{R}}_{B}\;I\left({\bar{R}}_{A},{\bar{R}}_{B}\right)},\; \end{array}$$

(44) $$\begin{array}{ccc} \lambda \bar{\eta }\left({\bar{R}}_{A},{\bar{R}}_{B}\right) & = & -\frac{1}{{\bar{R}}_{A}^{4}}+\frac{{\bar{R}}_{A}\sqrt{{\bar{R}}_{A}^{2}-1}+{\bar{R}}_{B}\sqrt{{\bar{R}}_{B}^{2}-1}}{{\bar{R}}_{A}{\bar{R}}_{B}\;I\left({\bar{R}}_{A},{\bar{R}}_{B}\right)}, \end{array}$$

where the function $I\left({\bar{R}}_{A},{\bar{R}}_{B}\right)\equiv {\bar{R}}_{A}\sqrt{{\bar{R}}_{B}^{2}-1}S\left({\bar{R}}_{A}\right)+{\bar{R}}_{B}\sqrt{{\bar{R}}_{A}^{2}-1}S\left({\bar{R}}_{B}\right)$. Substituting Equations (43) and (44) into Equation (42) obtains the distribution of the spontaneous curvature along the catenoid as the domain-renormalized function $\lambda {\bar{K}}_{0}\left(\bar{l};{\bar{R}}_{A},{\bar{R}}_{B}\right)$, defined in the interval $\bar{l}\in \left[-\sqrt{{\bar{R}}_{A}^{2}-1},\sqrt{{\bar{R}}_{B}^{2}-1}\right]$.

Equations (43) and (44), respectively, provide the constrictive radial force at the neck equator (necking waist constriction) and the axial force (a constant stretching), both in terms of the upper radii ${\bar{R}}_{A}$ and the lower radii ${\bar{R}}_{B}$ of the catenoid. Nevertheless, using Equation (40), it is possible to obtain an analytical expression for the radial force at any parallel in terms of the border radii and the arc length of the meridian generatrix. The constrictive force along the catenoid is given by

(45) $$\lambda F_{\rho }\left(\bar{l};{\bar{R}}_{A},{\bar{R}}_{B}\right)=-\frac{\bar{l}}{{\bar{R}}_{A}^{4}}+\frac{\lambda C({\bar{R}}_{A},{\bar{R}}_{B})}{\sqrt{1+{\bar{l}}^{2}}}-\frac{D({\bar{R}}_{A},{\bar{R}}_{B})}{\sqrt{1+{\bar{l}}^{2}}}\;\mathrm{arctanh}\left(\frac{\bar{l}}{\sqrt{1+{\bar{l}}^{2}}}\right),$$

where λC is given by Equation (43) and

(46) $$D\left({\bar{R}}_{A},{\bar{R}}_{B}\right)\equiv \frac{{\bar{R}}_{A}\sqrt{{\bar{R}}_{B}^{2}-1}+{\bar{R}}_{B}\sqrt{{\bar{R}}_{A}^{2}-1}}{{\bar{R}}_{A}{\bar{R}}_{B}\;I\left({\bar{R}}_{A},{\bar{R}}_{B}\right)}.$$

By evaluating Equation (45) at $\bar{l}=0$, we reproduce the necking force $F_{\rho }\left(0,{\bar{R}}_{A},{\bar{R}}_{B}\right)=C\left({\bar{R}}_{A},{\bar{R}}_{B}\right)$.

#### 4.1.1. Symmetric Solutions

**Figure 4:**
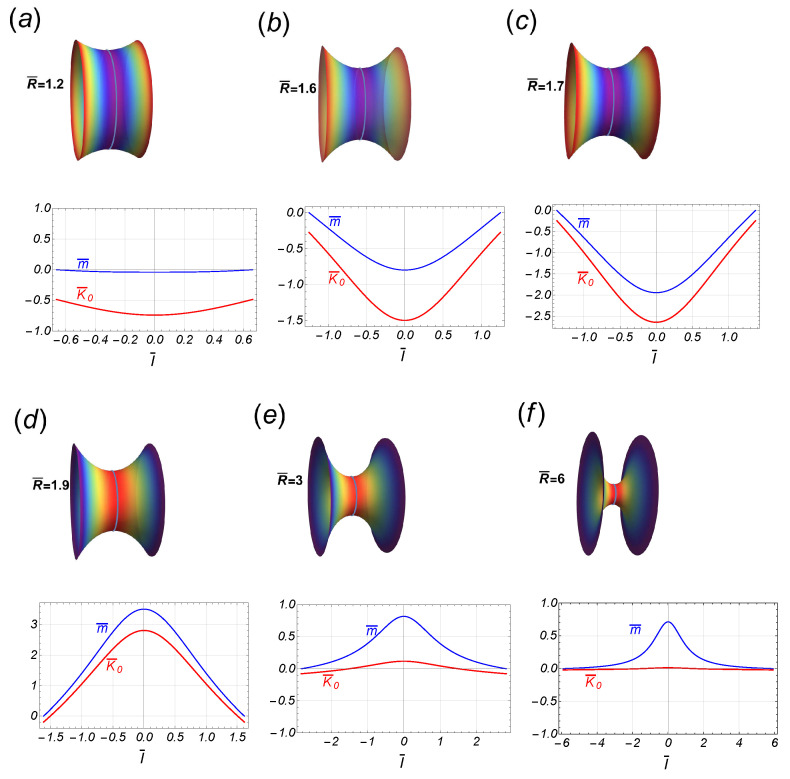
Some stages along the transition of the symmetric catenoidal shape. In the upper panel, the stages (**a**–**c**), are in the subcritical regime $\bar{R}<{\bar{R}}^{†}\approx 1.81$, while the stages in the bottom panel (**d**–**f**), are in the supercritical regime $\bar{R}>{\bar{R}}^{†}$. The corresponding behavior of the spontaneous curvature, $\bar{K}\left(\bar{l}\right)$, and the local torque $\bar{m}\left(\bar{l}\right)$, is shown as a function of the arc length $\bar{l}$. Note the flip-flop behavior of these functions as passing the critical value ${\bar{R}}^{†}$.

On a symmetric catenoid (defined as ${\bar{R}}_{A}={\bar{R}}_{B}=\bar{R}$), the necking force naturally equals zero, i.e., C=0, whereas the scaled axial force is $\lambda \bar{\eta }\left(\bar{R}\right)=-1/{\bar{R}}^{4}+1/\left[{\bar{R}}^{2}\;S\left(\bar{R}\right)\right];$ hence, in the symmetric neck, the scaled spontaneous curvature simplifies to $\lambda {\bar{K}}_{0}\left(\bar{l};\bar{R}\right)=S\left(\bar{l}\right)/\left[{\bar{R}}^{2}S\left(\bar{R}\right)\right]$. Because the function $S(\bar{R})$ is either positive (or negative) for $\bar{R}<{\bar{R}}^{†}$ (or $\bar{R}>{\bar{R}}^{†}$), we identify two different constrictional domains separated by a critical point (at $\bar{R}={\bar{R}}^{†}\approx 1.81$), which also describes the maximal area $\left({\bar{A}}_{max}\approx 1.19\right)$. In the subcritical regime ($\bar{R}<{\bar{R}}^{†}$), the function $S(\bar{R})$ decreases monotonically from unity down to zero (at $\bar{R}={\bar{R}}^{†}$). This necking mode makes the scaled spontaneous curvature inhomogeneously positive ($\lambda {\bar{K}}_{0}>0$) [4], which corresponds to an initially elongating neck appearing at predominantly cylindrical a surface (low constriction, leading neck stretching). At the critical point ($\bar{R}={\bar{R}}^{†}$), the symmetric critical catenoid exactly fulfills the geometric condition $S({\bar{R}}^{†})=0$ (also of maximal area). Although the constricted shape of the critical catenoid does not display any particular geometric feature, there are not analytic solutions for ${\bar{K}}_{0}$ and the other related properties in such a maximal area catenoid, i.e., there is a singularity in these inhomogeneous functions. In the supercritical regime $\left(\bar{R}>{\bar{R}}^{†}\right)$, however, the function $S(\bar{R})$ shifts to negative, hence making the inhomogeneous spontaneous curvature become negative ($\lambda {\bar{K}}_{0}<0$) in a predominantly concave neck geometry (high constriction, leading scissional pinching). In this regime, as $\bar{R}>>{\bar{R}}^{†}$, the spontaneous curvature is distributed almost homogeneously, making it zero asymptotically. Consequently, the axial force is positive in the subcritical regime ($\lambda \bar{\eta }>0$, if $\bar{R}<{\bar{R}}^{†}$) and negative in the supercritical regime ($\lambda \bar{\eta }<0$, if $\bar{R}>{\bar{R}}^{†}$). In both off-critical regimes, the boundary edge relationships are satisfied (Equation (18)), whereas at the critical point, these may be verified using L’Hopital’s rule. Note that at the catenoid waist, the scaled spontaneous curvature is given by $\lambda {\bar{K}}_{0}\left(0;\bar{R}\right)=1/\left[{\bar{R}}^{2}S\left(\bar{R}\right)\right]$, which takes large positive values if $\bar{R}$ approaches the critical point from the left ($\bar{R}\to {}^{-}{\bar{R}}^{†}$), whereas it takes large negative values approaching from the right ($\bar{R}\to {}^{+}{\bar{R}}^{†}$). If the symmetric function ${\bar{K}}_{0}\left(\bar{l}\right)$ is substituted in Equation (40), we find that the radial force, $-\bar{\gamma }\left(\bar{l}\right)$, is an odd function of the arc length $\bar{l}$, so that it vanishes at the waist of the neck and attains its maximal intensities at the border. In Figure 4, some stages of the necking pathway have been depicted for a particular case compatible with experimental rigidities (λ=1/(−0.7)). Notice the abrupt change in the inhomogeneous distribution of $\lambda {\bar{K}}_{0}$ as it passes through ${\bar{R}}^{†}$. Analogously, we can see the behavior of the local torque $\bar{m}$ (see Equation (41)) being negative in the subcritical region and positive in the supercritical region. That is, for catenoids in the subcritical region, the left side is pushing (and curving) the region to its right, while in the supercritical region, the right region is being pulled. As we can see, it is the waist of the catenoid where the most intense torque acts. In the quasi-cylindrical regime (very thick waist), the torque is practically zero since the contribution of the spontaneous curvature is cancelled out by the geometric contribution (i.e., $\bar{m}={\bar{K}}_{0}-1/\left(\lambda {\bar{\rho }}^{2}\right)\approx 0$). In contrast, in the thin catenoid regime, the spontaneous curvature is zero so that the torque is given by $\bar{m}\approx -1/\left(\lambda {\bar{\rho }}^{2}\right)$. Of course, at the border of the catenoid, the torque is zero due to the boundary conditions (see Figure 4).

By particularizing Equation (45) to the symmetric case (${\bar{R}}_{A}={\bar{R}}_{B}=\bar{R}$), the scaled radial force $F_{\rho }^{sym}\left(\bar{l}\right)\equiv F_{\rho }\left(\bar{l};R,R\right)$ is given by the odd function

(47) $$\lambda F_{\rho }^{sym}\left(\bar{l}\right)=-\frac{\bar{l}}{{\bar{R}}^{4}}-\frac{1}{{\bar{R}}^{2}S\left(\bar{R}\right)}\frac{1}{\sqrt{1+{\bar{l}}^{2}}}\mathrm{arctanh}\left(\frac{\bar{l}}{\sqrt{1+{\bar{l}}^{2}}}\right).$$

Further evaluation of this force on the lower border, $\bar{l}=\sqrt{{\bar{R}}^{2}-1}$, leads to the constrictional force in terms of the edge radii:

(48) $$\lambda F_{\rho }^{sym}\left(\bar{R}\right)=-\frac{\sqrt{{\bar{R}}^{2}-1}}{{\bar{R}}^{4}}+\frac{1}{{\bar{R}}^{2}\sqrt{{\bar{R}}^{2}-1}}\left(1-\frac{1}{S(\bar{R})}\right).$$

An isolated singularity appears at the critical point $\bar{R}={\bar{R}}^{†}\approx 1.81$. If $\bar{R}>>1$ (high constriction), we have $\lambda F_{\rho }^{sym}\propto -1/\left[{\bar{R}}^{3}\left(-1+\log\left(2\bar{R}\right)\right)\right]$, and thus, the radial force is attractive for large radii if λ>0; instead, if $\bar{R}\approx 1$ (low constriction), then $\lambda F_{\rho }^{sym}\propto -\sqrt{2}{\left(\bar{R}-1\right)}^{1/2}$, so that in this regime, the radial force is repulsive if λ<0.

More detailed information about the strength of the interaction can be obtained by numerically integrating the radial force. Particularly, the work *W* performed by the radial force $F_{\rho }\left({\bar{R}}_{B};{\bar{R}}_{A},{\bar{R}}_{B}\right)$ to evolve the lower border (from ${\bar{R}}_{B}=1$ to ${\bar{R}}_{B}>1$) obtains the associated energy, $U({\bar{R}}_{A},{\bar{R}}_{B})$, through

(49) $$W_{1\to {\bar{R}}_{B}}=\int_{1}^{{\bar{R}}_{B}}F_{\rho }\left({\bar{R}}^{\prime };{\bar{R}}_{A},{\bar{R}}^{\prime }\right)\;d{\bar{R}}^{\prime }\equiv -U\left({\bar{R}}_{A},{\bar{R}}_{B}\right).$$

An interesting behavior emerges from the singularity in the radial force. Close the singular point ${\bar{R}}^{†}$, the function form vanishes linearly as $S\left(\bar{R}\right)\approx -0.79\left(\bar{R}-{\bar{R}}^{†}\right)$, so that the corresponding singularities in *W* are cancelled out.

**Figure 5:**
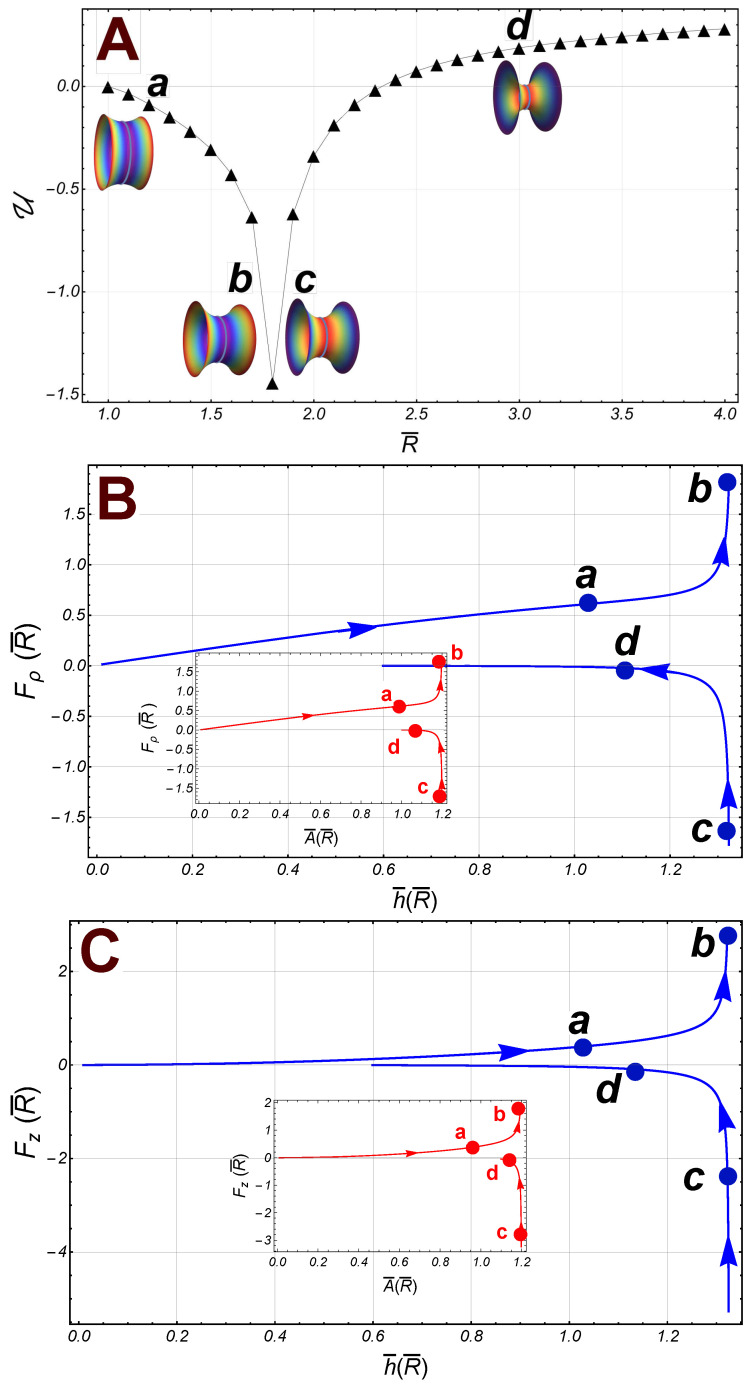
(**A**): The energy of the radial interaction $U(\bar{R})$ (black triangles) between the boundaries of a symmetric catenoid (we have taken the experimental reference λ=−1/0.7). Some catenoidal points of reference along the path: a ($\bar{R}=1.2$), b ($\bar{R}=1.7$), c ($\bar{R}=1.9$), and d ($\bar{R}=3$). The first two points belong to the subcritical regime, and the last two points are in the supercritical sector. The critical point is at ${\bar{R}}^{*}\approx 1.81$. (**B**): The radial force $F_{\rho }$, on the lower border (right), as a function of $\bar{h}$ (the separation distance between the borders). The inset panel outline the radial force as a function of the area of the catenoid. (**C**): The axial force $F_{z}\equiv -\bar{\eta }$ as a function of $\bar{h}$ (the distance of separation between the borders). The inset panel shows the axial force as a function of the area $\bar{A}$.

By attending to this necking work as a change of potential energy in the symmetric case here considered, the borders of the catenoid have no interaction for large radii (i.e., for $\bar{R}>>1$). The numerical result is the energy function, U, as shown in Figure 5A as black triangles (we have taken λ=−1/0.7). Let us notice the presence of a global minimum at the critical point ${\bar{R}}^{†}$. With regards to the corresponding forces, in the subcritical sector $\bar{R}<{\bar{R}}^{†}\approx 1.81$, the radial force on the border ${\bar{R}}_{B}$, is repulsive from the axial axis, whereas the axial force is attractive in this sector, i.e., $F_{z}<0$ (see Figure 5C, pathway points a and b). In approaching the critical point ($\bar{R}\to {\bar{R}}^{†}$), both the area, $\bar{A}$, and the height, $\bar{h}$, increase until reaching the size of the maximum catenoid such that ${\bar{A}}^{†}\approx 1.19$ and ${\bar{h}}^{†}\approx 1.32$; both the radial and the axial force diverge at this point (see Figure 5B,C). In the supercritical regime (${\bar{R}}^{†}<\bar{R}$), the radial force is attractive, and the lower border is repelled out with an axial force that tends to zero very quickly (see Figure 5C). Finally, a relaxation regime appears at large constrictions leading to final pinching scission; in this sector, the radial force is repulsive and decreasing with $\bar{R}$, tending to zero for a very thin catenoid, i.e., such that $\bar{R}>>1$. Thus, in this abscissional limit, the boundaries do not interact each other, although the separation decreases strongly ($\bar{h}\to 0$) and the area $\bar{A}\to 1$ (see Figure 5B,C, pathway points c and d).

#### 4.1.2. Asymmetric Solutions

Let us now analyze the general asymmetric case such that the upper border is fixed to ${\bar{R}}_{A}>1$ and ${\bar{R}}_{A}\neq {\bar{R}}_{B}$. The axial force is still given by Equation (44), so that if the lower border ${\bar{R}}_{B}>>1$, then the axial force $F_{z}\equiv -\bar{\eta }\propto 1/\left(\lambda {\bar{R}}_{A}^{4}\right)$. Hence, if λ<0, the lower edge is repelled out from the upper one; in contrast, if λ>0, the lower border is conversely attracted thereby. Further, the radial force on the lower border is given by Equation (45); if substituting $\bar{l}={\bar{l}}_{B}=\sqrt{{\bar{R}}_{B}^{2}-1}$, we found:

(50) $$\lambda F_{\rho }\left({\bar{R}}_{B};{\bar{R}}_{A},{\bar{R}}_{B}\right)=-\frac{\sqrt{{\bar{R}}_{B}^{2}-1}}{{\bar{R}}_{A}^{4}}+\frac{\lambda C({\bar{R}}_{A},{\bar{R}}_{B})}{{\bar{R}}_{B}}-\frac{D({\bar{R}}_{A},{\bar{R}}_{B})}{\sqrt{{\bar{R}}_{B}^{2}-1}}\;\left(1-S({\bar{R}}_{B})\right).$$

**Figure 6:**
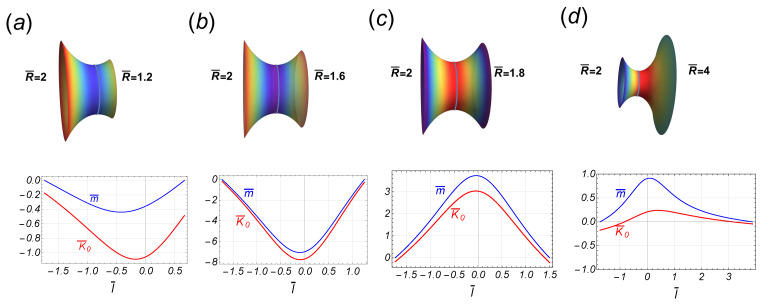
Some stages along an asymmetric transition, such that the upper (left) radii is fixed to be ${\bar{R}}_{A}=2$. The critical point occurs at ${\bar{R}}^{*}\approx 1.65$, so that the first two stages (**a**,**b**), are in the subcritical regime while the last two (**c**,**d**), belong to the supercritical regime. Note that both functions, ${\bar{K}}_{0}\left(\bar{l}\right)$ and $\bar{m}\left(\bar{l}\right)$, are asymmetric functions along the corresponding catenoid.

In the regime of high constriction (${\bar{R}}_{B}>>1$), then $F_{\rho }\left({\bar{R}}_{B}\right)\propto -{\bar{R}}_{B}/\left(\lambda {\bar{R}}_{A}^{4}\right)$, so that the radial force on the lower border is repulsive from the axial axis if λ<0. Despite the absence of mirror symmetry in this case, the radial force (50) also presents a singularity at the point ${\bar{R}}_{B}={\bar{R}}_{B}^{*}$, determined by the condition $I({\bar{R}}_{A},{\bar{R}}_{B}^{*})=0$. For the reference result (at λ=−1/0.7), the behavior of the spontaneous curvature close to the critical point has been depicted in Figure 6, in the case of fixed ${\bar{R}}_{B}=2$ and critical point ${\bar{R}}_{B}^{*}\approx 1.65$.

In the subcritical regime (see Figure 6a,b), both ${\bar{K}}_{0}\left(\bar{l}\right)$ and $\bar{m}\left(\bar{l}\right)$ are negative functions; in the supercritical regime, they are positive. The torque vanishes on the boundaries but is not a symmetric function. Thus, in the subcritical regime, the left side of the catenoid is pushing the right side and curving it more intensely at the waist (i.e., $\bar{l}=0$), while in the supercritical regime, the right side is pulled (see Figure 6c,d).

#### 4.1.3. The Fixed Radii, ${\bar{R}}_{A}=1$

If the upper border is fixed to be ${\bar{R}}_{A}=1$, then we have that $S(\bar{R}=1)=1$, so that the necking force $\lambda C\left(1,{\bar{R}}_{B}\right)=\left[1-{\bar{R}}_{B}^{2}S\left({\bar{R}}_{B}\right)\right]/\left[{\bar{R}}_{B}\sqrt{1-{\bar{R}}_{B}^{2}}\right]$ and $D\left(1,{\bar{R}}_{B}\right)=1/{\bar{R}}_{B}$. Thus, the axial force $\bar{\eta }=0$ and the spontaneous curvature are given by

(51) $$\lambda K_{0}\left(\bar{l};1,{\bar{R}}_{B}\right)=S\left(\bar{l}\right)+\frac{\left(1-{\bar{R}}_{B}^{2}S\left({\bar{R}}_{B}\right)\right)}{{\bar{R}}_{B}\sqrt{{\bar{R}}_{B}^{2}-1}}\frac{\bar{l}}{\sqrt{1+{\bar{l}}^{2}}},$$

where $\bar{l}\in \left[0,\sqrt{{\bar{R}}_{B}^{2}-1}\right]$. As a function of ${\bar{R}}_{B}$, Equation (51) does not show any singularity. On the lower boundary, the radial force is given by

(52) $$\lambda F_{\rho }\left({\bar{R}}_{B};1,{\bar{R}}_{B}\right)=-\sqrt{{\bar{R}}_{B}^{2}-1}-\frac{{\bar{R}}_{B}-1}{{\bar{R}}_{B}\sqrt{{\bar{R}}_{B}^{2}-1}}\left(\frac{1}{{\bar{R}}_{B}}+S\left({\bar{R}}_{B}\right)\right).$$

This force does not present any singularity either; it vanishes if ${\bar{R}}_{B}=1$. For ${\bar{R}}_{B}\approx 1$, we have $F_{\rho }\left({\bar{R}}_{B}\right)\propto -\left(2\sqrt{2}/\lambda \right)\sqrt{{\bar{R}}_{B}-1}$, and $F_{\rho }\left({\bar{R}}_{B}\right)\propto -{\bar{R}}_{B}/\lambda$, if ${\bar{R}}_{B}>>1$. Thus, the radial force is attractive if λ>0 and repulsive if λ<0. The local torque (41) can be written as $\lambda \bar{m}\left(\bar{l}\right)=\lambda {\bar{K}}_{0}\left(\bar{l}\right)-1/\left(1+{\bar{l}}^{2}\right)$. On the boundary, the torque vanishes as a consequence of the boundary condition Equation (18).

**Figure 7:**
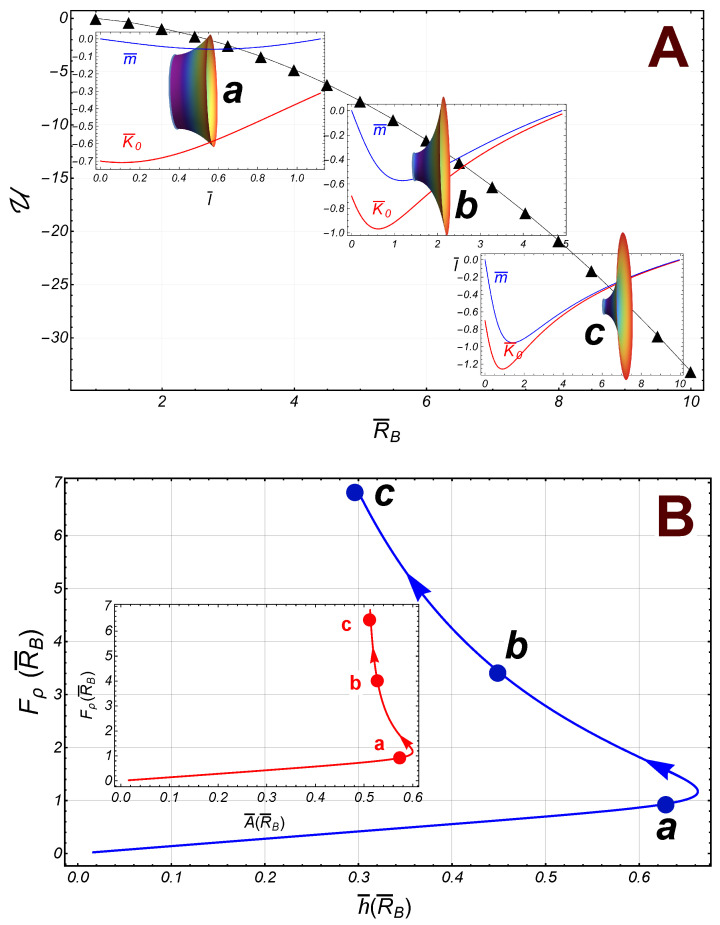
Numerical results in the case of fixed radii ${\bar{R}}_{A}=1$ and 1/λ=−0.7. (**A**): The energy of interaction U (black triangles) between the borders as a function of the radii ${\bar{R}}_{B}$. As reference, some catenoidal points have been identified along the way: $a({\bar{R}}_{B}=1.5)$, $b({\bar{R}}_{B}=5)$, and $c({\bar{R}}_{B}=10)$. The distribution of the curvature ${\bar{K}}_{0}$ and the torque $\bar{m}$ have been depicted at the corresponding inset panel. (**B**): The radial force $F_{\rho }$, on the lower border as a function of $\bar{h}$, the distance of separation between the borders. The inset panel shown the radial force as a function of the area $\bar{A}$. The catenoid of maximal size reaches at ${\bar{R}}_{B}^{†}\approx 1.81$. Note that asymptotically, the area $\bar{A}\to 1/2$.

Some numerical results have been displayed in Figure 7 (we have taken 1/λ=−0.7). In contrast with the symmetric case, the energy U is a decreasing smooth function of the radii ${\bar{R}}_{B}$, so that the radial force on the lower border (the right border in the Figure 7A) is also increasing. Both the spontaneous curvature and the local torque are negative functions along the catenoid. It is the torque that causes the curvature of the catenoid; since it is negative, the left sector pushes the right one (see the inset catenoids in Figure 7A). Further, for small values of ${\bar{R}}_{B}$, the area, $\bar{A}$, increases until it reaches its maximum value at the point ${\bar{R}}^{†}\approx 1.81$; then, it begins to decrease such that if ${\bar{R}}_{B}>>1$, the area $\bar{A}\to 1/2$; similarly, the separation between the borders, $\bar{h}$, reaches its maximum value at ${\bar{R}}^{†}$; then, it decreases further. finally, in the asymptotic limit, $\bar{h}\to 0$ (see Figure 7B).

## 5. Discussion

In our quasi-static approach to the neck shaping process, we have implemented sequences of static curvature configurations for a CH inhomogeneous fluid membrane considering a flexible sheet in two dimensions with no dissipation explicitly considered [39]. Our inhomogeneous CH model recapitulates membrane complexities under the mesoscopic concept that we refer to as “fluid mosaicity”—with reference to the widely accepted fluid mosaic model of biological membranes [14]. It is worthy remember that any shape deformation process within the CH field is essentially adiabatic (thus subsidiarily inviscid) as it only involves elastic free energy but no heat exchange (as reviewed in ref. [43]). Hence, the necking sequences considered stationary here describe an ideal (frictionless) class of static deformations expending free energy at an internal mechanical equilibrium with the CH field of curvature elasticity. Indeed, the mechanical work involved in the CH shape changes has been ideally considered under reversible adaptation at equilibrium (conservative), but not as a transient process working out-of-equilibrium (dissipative). However, explicit entropy exchanges due to chemical reorganizations have not been considered either to describe possible molecular heterogeneity (as seminally described by Markin [57]; later, developed in a physicochemical basis by Szleifer et al. [48]; and further reviewed by Safran [44]). In a real biological necking process, the dynamic membrane architecture is based on an elastic transformation and lateral redistribution of components into the curvature shapes as primarily driven by chemical, physical and geometric gradients [19]. Such complexities are usually approached from a thermodynamic equilibrium standpoint as a composition–curvature coupling in which each particular membrane configuration is assumed to be in reversible equilibrium with a heterogeneous lipid reservoir, both considered to be chemically closed (recently reviewed by Bashkirov et al. [5]). Despite the powerfulness of the equilibrium thermodynamics tool, realistic descriptions of biochemical heterogeneity within a dynamical necking scenario, in practice, are quite impracticable, as it requests in considering a fairly big changing number of lipids and proteins (chemically opened). Instead, we have considered a more simplistic approach in which the dynamic heterogeneity of biological necking is effectively captured under “fluid mosaicity” conditions by potential deformation effectors as being locally inhomogeneous, i.e., we allow the spontaneous curvature and the components of an anisotropic membrane tension to vary spatially to minimize the deformation work under thermodynamically effective adiabatic and isochemical conditions actually “hidden” in the CH field of curvature elasticity. Our mesoscopic 2D-continuous analytics has been performed in a similar way as the elasticity theory of thin plates, but considering rotational symmetry as a sine qua non condition requested by macroscopic fluidity [39]. Of course, no explicit account of the internal microstructure is considered by our CH approach, which considers laterally inhomogeneous conditions. In a molecular context, Safran, Szleifer and coworkers have considered the mesoscopic inhomogeneities being recapitulated under lateral dependencies of internal pressure profiles along the membrane thickness [44,48]. The thermostatistical integrals for the CH parameters manifest essential mosaic inhomogeneity due to microstructural forces beneath the lipid bilayer [49]. Specifically, an inhomogeneous spontaneous curvature occurs due to transverse bilayer asymmetries, e.g., compositional, geometric, electrostatic, leaflet thickness, etc. [43,45]. These microphysical complexities have been coarse-grained in our CH approach under spatial inhomogeneities of the local mechanical effectors of curvature: specifically, the local values of spontaneous curvature ($K_{0}$) and the requested anisotropic values of the necking tensions (η,γ), respectively, for axial stretching and radial constriction. We expect the locally inhomogeneous values of spontaneous curvature and constriction tension to represent anisotropic (mosaic) remodeling forces, i.e., they represent membrane mosaicities naturally imparting necking order in the mesoscale.

In the first part of this work, based on the inhomogenous Canham–Helfrich model settled for a laterally modulable distribution of mechanical effectors (specifically, variable $K_{0}$ and γ, and constant η), we have established the equilibrium shape equation as being an Euler–Lagrange minimizer with the addition that the spontaneous curvature and the constrictional tension are inhomogeneous functions along the axial membrane coordinate. The constant bending rigidities for pure splay (κ) and saddle-splay ($\kappa_{G}$) represent isotropically fluid forces homogeneously deployed along the membrane necking shape as far they determine the macroscopic scale of bending energy imposed by the global neck topology (κ determines the global neck rigidness, and $\kappa_{G}$ determines the total strength of the cortical forces injected in the neck waist from the border). In more technical words, they constitute the intensive densities of elastic resistance (flexibility) isotropically fixed under mesoscopic fluidity. This is the flexible fluidness property invoked by W. Helfrich in his foundational formulation of the model [39]. In this mesoscopic perspective, we consider the ideal (adiabatic) case so that any entropic interaction has been neglected: the local value of spontaneous curvature is the essential factor that determines the energetically optimal necking configuration. The inhomogeneous values of the spontaneous curvature should reflect possible microscopic asymmetries in the lipid bilayer [39]; these can occur through different mechanisms of curvature remodeling, for instance, molecular shape effectors [20,21,22,23,24,25,26] and compositional differences [5,30,45,48], among others. These local curvature effectors request each leaflet to adjust its area so that the bilayer bends at each site in some preferential side. Thus, the local torque involved in the bending emerges from the localized spontaneous curvature that depends on the mosaic distribution of the membrane components. Of course, if the membrane components differently concentrate in one of the sides with respect to the other, e.g., driven under differential cortical flows (nonlubricated), then, the membrane bends again to reach a relaxed configuration due to the difference in the accumulated area in each side [45]. Although the inhomogenous CH model captures these local asymmetries in a spatial variable $K_{0}$, we have demonstrated the necessity to consider additional energetic necking terms associated with local constriction under global stretching. Hence, we have obtained the mechanical information emerging from inhomogeneities in $K_{0}$ and related γ, which is discussed below in terms of mesoscopic interactions and criticality. However, how these results are related to the corresponding microscopic (molecular) interactions remains to be investigated.

In the second part of our work, we capture the effects of those mechanical necking inhomogeneities superposed to the catenoid membrane shape, a minimal surface with an average zero value of mean curvature which recapitulates “homeostatic” conditions (being adiabatically stable and energetically minimal). Thus, we have analyzed the boundary conditions imposing anisotropic necking tensions, the distribution of ${\bar{K}}_{0}$ and the torque itself, as well as the elastic forces such that the membrane necking shape is adapted to a mechanically mosaic (inhomogeneous) catenoid. Except the case with fixed ${\bar{R}}_{A}=1$, the catenoidal solutions belong to two possible branches separated by the critical catenoid, which is reached for some value ${\bar{R}}_{B}={\bar{R}}_{B}^{*}$; it is identified as a critical point in the sense that it signals an abrupt change in the behavior of the spontaneous curvature. In the symmetric case, such that the two radii borders are equal, say $\bar{R}$, the critical catenoid and the catenoid of maximum size are the same, reached for ${\bar{R}}^{*}\approx 1.81$. In addition, if we assume the experimental result $\kappa_{G}/\kappa =-0.7$, widely admitted for the configurations of fluid biological membranes and vesicle models [43], the energy of interaction between the borders attains its global minimum at the critical point ${\bar{R}}^{*}$. Thus, the radial force is repulsive between the borders for thick catenoidal shapes, attractive in the regime of thin catenoids, and, for large radii, the borders do not interact each other. We envisage this critically inhomogeneous interaction setting to theoretically capture much of the most essential features of biological necking. In particular, the distribution of the spontaneous curvature ${\bar{K}}_{0}$ and the torque $\bar{m}$ has been depicted in Figure 4 and Figure 6 in the symmetric case and the asymmetric one (for fixed ${\bar{R}}_{A}=2$), respectively. The curved shape of the catenoid is the effect of the torque $\bar{m}$. In the quasi-cylindrical region, it is practically zero, although its negativity implies that the region to the left pushes the opposite region to its right. Near the critical point, in the subcritical region, the torque remains negative and is very intense at the waist of the catenoid and abruptly jumps to be positive when it passes to the supercritical region (see Figure 4 and Figure 6). In the very thin catenoidal regime, where ${\bar{K}}_{0}$ vanishes, the normal curvature of the waist is the only contribution to the local torque. Such a sequence of static necking configurations seems to essentially recapitulate the different steps of furrow constriction and scissional pinching observed to be driven by the mitotic spindle in eukaryote cytokinesis [1,2,3]. Also, the necking criticality assumed to be inhomogeneous in our work could be assimilated to explore the complex dynamics driven by dynamin proteins in biological organelles [7,58]. Previously, the presence of bistable criticality in organelle necks has been modeled as composite homogeneous solutions joining a scissional tube to two half-catenoid hemispheres [6].

Despite our reductionist idealizations of the necking process as a minimally functional adiabatic process, however, biological necking proceeds out-of-equilibrium, i.e., budding, cell division, fission, etc., which are naturally irreversible processes. The chemical potentials (and the total entropy) can vary by exchanging some heat and materials between the membrane neck and its biochemical surroundings. Of course, our adiabatic/isochemical necking CH model resolved under minimal catenoid solution does not constitute an exact nor detailed description of the real biological processes; however, it could reasonably capture the biological idea of homeostatic fitness under instantaneous mechanical equilibrium [36]. Hence, our reductionism recapitulates some realistic conditions of the biological system, in particular, adaptative slowness (staticity) and practical absence of frictional dissipation (fluidity), both leading to physical “adiabatic stability” compatible with physiological homeostasis [42]. Indeed, our inhomogeneous CH model implements effective adiabatic conditions as an optimal (conservative) expenditure of elastic free energy for neck shape reorganization. The CH model does not make explicit biochemical changes requested under fluid deformation. It also does not have entropy creation related to chemical change [39,43]. However, heterogeneous chemical contributions appear to effectively embedded within the membrane tension anisotropy, as far the inhomogeneous function sigma recapitulates a spatially inhomogeneous density of membrane energy [5,44,48,57]. Therefore, the resulting quasi-static sequences of necking CH shapes obtained under catenoid-like constriction in the different scenarios are expected to be “homeostatic” [36]; in other words, “adiabatically stable” is regarded as energetically optimal (no heat exchange involved), i.e., not exhibiting dissipation in a near-reversible (frictionless) succession of mechanically equilibrated fluid states [42]. More realistic necking descriptions would implement irreversibility within viscous friction terms, making an explicit generation of dissipative entropy under finite necking rate (as in, e.g., [59,60]). However, these dynamic complexities go against the “conservatively homeostatic” definition of fluid reversibility inherent to the considered reductionism under a mechanical Canham–Helfrich necking equilibrium.

A thermodynamically and kinetically more complete description of homeostatic irreversibility should eventually go beyond the conservative CH field here exploited for the quasi-static description of the membrane necking configurations. Newer theoretical and computational approaches to necking kinetics could be implemented around the concept of CH gradient flow (first introduced in [61]). Such further extensions would include dissipative friction accounting for the possible entropy generations characteristic of the real necking system. The results presented in this work focus on catenoidal necking shapes as mostly observed in cellular processes of eukaryote cytokinetic scission such as cell division [1,2,3] and endo/exocytosis [10,11,12,13]. However, previous studies on organelle dynamics suggest extending the analysis beyond catenoidal shapes to other complex structures as in the intracellular transport through membrane tubules [6,7] or to take into account the dynamically asymmetric stresses transmitted by dynamin proteins which play a relevant role in the organelle fission [58].

## 6. Summary and Conclusions

By generalizing the CH energy to the case with inhomogeneous spontaneous curvature (${\bar{K}}_{0}$) and surface tension ($\bar{\sigma }$), in this theoretical work, we have found:

1. Local inhomogeineity (mechanical mosaicity). In addition to the inhomogeneous membrane shape equation, the geometric connection Equation (8) must be also satisfied. They locally relate the inhomogeneities in σ and ${\bar{K}}_{0}$ and constitute the conditions of mechanical equilibrium that determine the constrictive and stretching forces requested for a given necking configuration under optimizing inhomogeneity.
2. Modulo boundary conditions: the shape membrane is a catenoid under necking, if and only if the spontaneous curvature is given by the analytical formula given by Equation (42). This constitutive equation represents a strict necking condition established between the constrictive and stretching forces requesting a lateral distribution of $K_{0}$ for given boundary conditions (fixing dimensionless constriction as a border-to-waist relative radius). This contributes to fixing the local torque as given by Equation (41).
3. The equilibrium catenoidal configurations belong to two branches separated by a critical catenoid. In the symmetrical case, the critical catenoid corresponds to the one of maximum size (the same found from the soap film analysis). In the subcritical regime, both the torque and ${\bar{K}}_{0}$ are negative functions (leading predominant thick catenoid representing initial constriction), while they become positive functions in the supercritical regime, leading to predominant concavity (representing the final pinching regime). ${\bar{K}}_{0}$ is practically zero in the very thin catenoidal regime.
4. Analytical formulas corresponding to the axial stretching elastic force and the constrictive force are given by Equations (44) and (45), respectively. These are general results, whether the catenoid is symmetric or asymmetric. These results show that in the thick catenoid sector, the radial interaction between the boundaries is repulsive, whereas it is attractive in the regime of thin catenoids; in the sector of very thin catenoidal shapes, the interaction vanishes.
  We summarize our conclusions from a biological perspective:
5. The inhomogeneous CH model here described implements effective adiabatic conditions as an optimal (conservative) expenditure of elastic free energy for neck shape reorganization. Although our CH model neither makes explicit biochemical change requested under fluid deformation nor entropy creation related to chemical change, the heterogeneous chemical potentials involved in biological necking are effectively embedded within anisotropic membrane tension inhomogeneities as far they recapitulate the spatially variable density of membrane energy.
6. The resulting sequences of inhomogeneous necking CH configurations are implicitly “homeostatic” in the sense of being adiabatically stable and energetically optimal (no heat exchange involved).
7. More realistic necking descriptions would implement irreversibility within frictional terms, making an explicit generation of dissipative entropy under finite necking rate. However, these dynamic complexities would be formulated at compatibility with the biological principle of ”conservative homeostasis”, which is currently captured (in maximalism) by the considered reductionism under mechanical Canham–Helfrich necking equilibrium.
